# *cis*-Bis(2,2′-bipyrimidine-κ^2^ *N* ^1^,*N* ^1′^)di­iodidomanganese(II)

**DOI:** 10.1107/S160053681105080X

**Published:** 2011-12-03

**Authors:** Kwang Ha

**Affiliations:** a School of Applied Chemical Engineering, The Research Institute of Catalysis, Chonnam National University, Gwangju 500-757, Republic of Korea

## Abstract

The asymmetric unit of the title complex, [MnI_2_(C_8_H_6_N_4_)_2_], contains one half of a neutral Mn^II^ complex, with the entire molecule completed by the application of twofold symmetry. The Mn^II^ ion is six-coordinated in a distorted octa­hedral environment defined by four N atoms of the two chelating 2,2′-bipyrimidine (bpym) ligands and two I^−^ anions in a *cis*-N_4_I_2_ coordination geometry. The dihedral angle between the least-squares planes of the two bpym ligands (r.m.s deviation for all non-H atoms = 0.063 Å) is 85.04 (6)°. In the crystal, complex mol­ecules are connected by C—H⋯N and C—H⋯I hydrogen bonds, forming a three-dimensional network. Mol­ecules are stacked in columns along the *a* axis. Along the *c* axis, successive mol­ecules stack in the opposite directions.

## Related literature

For related crystal structures of [MnI(bpym)_2_(H_2_O)]I·*x*H_2_O (*x* = 2, 1), see: Ha (2011*a* ▶,*b* ▶).
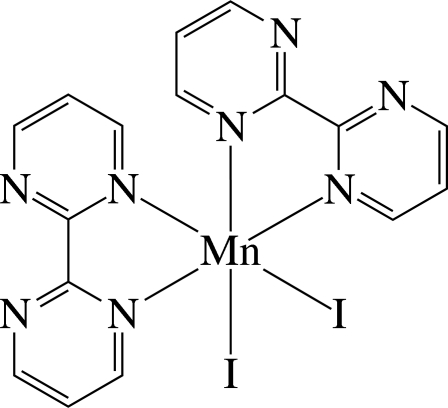


## Experimental

### 

#### Crystal data

[MnI_2_(C_8_H_6_N_4_)_2_]
*M* *_r_* = 625.08
Monoclinic, 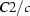

*a* = 8.2841 (4) Å
*b* = 13.8442 (7) Å
*c* = 17.8243 (9) Å
β = 97.822 (1)°
*V* = 2025.19 (17) Å^3^
*Z* = 4
Mo *K*α radiation
μ = 3.72 mm^−1^
*T* = 200 K
0.34 × 0.22 × 0.17 mm

#### Data collection

Bruker SMART 1000 CCD diffractometer
Absorption correction: multi-scan (*SADABS*; Bruker, 2000 ▶) *T*_min_ = 0.863, *T*_max_ = 1.000
7130 measured reflections
2477 independent reflections
1923 reflections with *I* > 2σ(*I*)
*R*_int_ = 0.023

#### Refinement

*R*[*F* ^2^ > 2σ(*F* ^2^)] = 0.027
*wR*(*F* ^2^) = 0.064
*S* = 1.10
2477 reflections
123 parameters
H-atom parameters constrained
Δρ_max_ = 0.94 e Å^−3^
Δρ_min_ = −0.50 e Å^−3^

### 

Data collection: *SMART* (Bruker, 2000 ▶); cell refinement: *SAINT* (Bruker, 2000 ▶); data reduction: *SAINT*; program(s) used to solve structure: *SHELXS97* (Sheldrick, 2008 ▶); program(s) used to refine structure: *SHELXL97* (Sheldrick, 2008 ▶); molecular graphics: *ORTEP-3* (Farrugia, 1997 ▶) and *PLATON* (Spek, 2009 ▶); software used to prepare material for publication: *SHELXL97*.

## Supplementary Material

Crystal structure: contains datablock(s) global, I. DOI: 10.1107/S160053681105080X/bt5729sup1.cif

Structure factors: contains datablock(s) I. DOI: 10.1107/S160053681105080X/bt5729Isup2.hkl

Additional supplementary materials:  crystallographic information; 3D view; checkCIF report

## Figures and Tables

**Table:** 

Mn1—N4	2.289 (3)
Mn1—N1	2.305 (3)
Mn1—I1	2.8410 (5)

**Table:** 

N4^i^—Mn1—N1	71.78 (10)
I1—Mn1—I1^i^	104.19 (3)

Symmetry code: (i) 
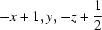
.

**Table 2:** Hydrogen-bond geometry (Å, °)

*D*—H⋯*A*	*D*—H	H⋯*A*	*D*⋯*A*	*D*—H⋯*A*
C6—H6⋯N3^ii^	0.95	2.60	3.151 (5)	117
C6—H6⋯N2^iii^	0.95	2.62	3.554 (5)	166
C7—H7⋯I1^iv^	0.95	2.97	3.917 (4)	173

Symmetry codes: (ii) 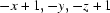; (iii) 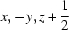; (iv) 
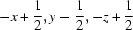
.

**Fig. 1.**
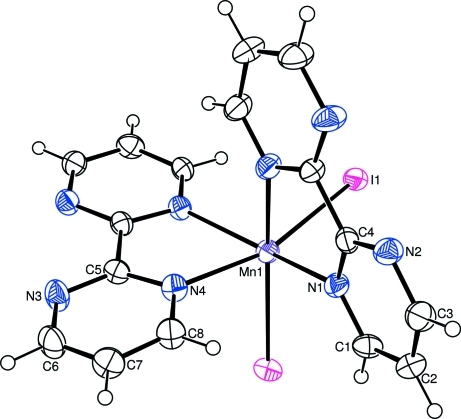
The structure of the title complex, with displacement ellipsoids drawn at the 40% probability level for non-H atoms. Unlabelled atoms are related to the reference atoms by the (1 - x, y, 1/2 - z) symmetry transformation.

**Fig. 2.**
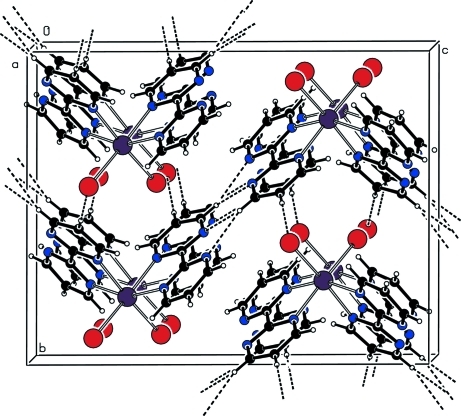
View of the unit-cell contents of the title complex. Hydrogen-bond interactions are drawn with dashed lines.

## supplementary crystallographic information

### Comment

The asymmetric unit of the title complex, [MnI_2_(bpym)_2_] (bpym = 2,2'-bipyrimidine, C_8_H_6_N_4_), contains one half of a neutral Mn^II^ complex (Fig. 1). The complex is disposed about a twofold rotation axis running in the [010] direction passing through the Mn1 atom. The Mn^II^ ion is six-coordinated in a distorted octahedral environment defined by four N atoms of the two chelating bpym ligands and two I^-^ anions in a *cis*-N_4_I_2_ coordination geometry. The structure is quite different from the previously reported complexes [MnI(bpym)_2_(H_2_O)]I.xH_2_O obtained from a methanol (*x* = 2; Ha, 2011*a*) or a 2-butanone solution (*x* = 1; Ha, 2011*b*) of the same reaction product, in which two bpym ligands, an I^-^ anion and a water ligand are coordinated to the Mn^II^ ion, respectively.

The tight N—Mn—N chelating angles and I—I repelling (Table 1) contribute the distortion of ocataheron, which results in non-linear *trans* axes (<N1—Mn1—N1^i^ = 156.60 (15)° and <I1—Mn1—N4 = 164.40 (8)°; symmetry code i: 1 - *x*, *y*, 1/2 - *z*). Because the Mn—N bond lengths are nearly equivalent (Table 1), the different *trans* effects of the I and N atoms cannot be observed reliably. The dihedral angle between the least-squares planes of the two bpym ligands [maximum deviation = 0.098 (3) Å] is 85.04 (6)°. In the crystal, the complex molecules are connected by intermolecular C—H···N and C—H···I hydrogen bonds, forming a three-dimensional network (Fig. 2, Table 2). Molecules are stacked in columns along the *a* axis. When viewed down the *c* axis, successive molecules stack in the opposite directions. In the columns, several inter- and intramolecular π-π interactions between adjacent pyrimidine rings are present, the shortest ring centroid-centroid distance being 3.853 (2) Å.

### Experimental

To a solution of 2,2'-bipyrimidine (0.1587 g, 1.003 mmol) in acetone (40 ml) was added MnI_2_ (0.1540 g, 0.499 mmol) and refluxed for 3 h. The formed precipitate was separated by filtration, washed with acetone and dried at 50 °C, to give a yellow powder (0.0701 g). Crystals suitable for X-ray analysis were obtained by slow evaporation from a dimethyl sulfoxide (DMSO) solution at 90 °C.

### Refinement

H atoms were positioned geometrically and allowed to ride on their respective parent atoms [C—H = 0.95 Å and *U*_iso_(H) = 1.2*U*_eq_(C)]. The highest peak (0.94 e Å^-3^) and the deepest hole (-0.50 e Å^-3^) in the difference Fourier map are located 1.35 Å and 0.83 Å from the atoms N3 and C2, respectively.

### Crystal data

**Table:**

[MnI_2_(C_8_H_6_N_4_)_2_]	*F*(000) = 1180
*M**_r_* = 625.08	*D*_x_ = 2.050 Mg m^−^^3^
Monoclinic, *C*2/*c*	Mo *K*α radiation, λ = 0.71073 Å
Hall symbol: -C 2yc	Cell parameters from 3917 reflections
*a* = 8.2841 (4) Å	θ = 2.9–28.1°
*b* = 13.8442 (7) Å	µ = 3.72 mm^−^^1^
*c* = 17.8243 (9) Å	*T* = 200 K
β = 97.822 (1)°	Block, yellow
*V* = 2025.19 (17) Å^3^	0.34 × 0.22 × 0.17 mm
*Z* = 4	

### Data collection

**Table:**

Bruker SMART 1000 CCD diffractometer	2477 independent reflections
Radiation source: fine-focus sealed tube	1923 reflections with *I* > 2σ(*I*)
graphite	*R*_int_ = 0.023
φ and ω scans	θ_max_ = 28.3°, θ_min_ = 2.3°
Absorption correction: multi-scan (*SADABS*; Bruker, 2000)	*h* = −10→11
*T*_min_ = 0.863, *T*_max_ = 1.000	*k* = −18→18
7130 measured reflections	*l* = −23→13

### Refinement

**Table:**

Refinement on *F*^2^	Primary atom site location: structure-invariant direct methods
Least-squares matrix: full	Secondary atom site location: difference Fourier map
*R*[*F*^2^ > 2σ(*F*^2^)] = 0.027	Hydrogen site location: inferred from neighbouring sites
*wR*(*F*^2^) = 0.064	H-atom parameters constrained
*S* = 1.10	*w* = 1/[σ^2^(*F*_o_^2^) + (0.0197*P*)^2^ + 5.0272*P*] where *P* = (*F*_o_^2^ + 2*F*_c_^2^)/3
2477 reflections	(Δ/σ)_max_ = 0.001
123 parameters	Δρ_max_ = 0.94 e Å^−^^3^
0 restraints	Δρ_min_ = −0.50 e Å^−^^3^

### Special details

**Table:**

Geometry. All e.s.d.'s (except the e.s.d. in the dihedral angle between two l.s. planes) are estimated using the full covariance matrix. The cell e.s.d.'s are taken into account individually in the estimation of e.s.d.'s in distances, angles and torsion angles; correlations between e.s.d.'s in cell parameters are only used when they are defined by crystal symmetry. An approximate (isotropic) treatment of cell e.s.d.'s is used for estimating e.s.d.'s involving l.s. planes.
Refinement. Refinement of *F*^2^ against ALL reflections. The weighted *R*-factor *wR* and goodness of fit *S* are based on *F*^2^, conventional *R*-factors *R* are based on *F*, with *F* set to zero for negative *F*^2^. The threshold expression of *F*^2^ > σ(*F*^2^) is used only for calculating *R*-factors(gt) *etc*. and is not relevant to the choice of reflections for refinement. *R*-factors based on *F*^2^ are statistically about twice as large as those based on *F*, and *R*- factors based on ALL data will be even larger.

### Fractional atomic coordinates and isotropic or equivalent isotropic displacement parameters (Å^2^)

**Table:**

	*x*	*y*	*z*	*U*_iso_*/*U*_eq_	
Mn1	0.5000	0.28328 (5)	0.2500	0.03038 (18)	
I1	0.68736 (3)	0.409360 (18)	0.170347 (15)	0.03768 (9)	
N1	0.2923 (4)	0.2495 (2)	0.15380 (16)	0.0316 (7)	
N2	0.2149 (4)	0.1486 (2)	0.04676 (18)	0.0372 (7)	
N3	0.4761 (4)	0.0785 (2)	0.4388 (2)	0.0444 (9)	
N4	0.4157 (4)	0.1610 (2)	0.32184 (18)	0.0349 (7)	
C1	0.1449 (5)	0.2901 (3)	0.1444 (2)	0.0359 (8)	
H1	0.1204	0.3392	0.1783	0.043*	
C2	0.0271 (5)	0.2616 (3)	0.0861 (2)	0.0421 (10)	
H2	−0.0780	0.2903	0.0789	0.051*	
C3	0.0689 (5)	0.1895 (3)	0.0387 (2)	0.0408 (9)	
H3	−0.0105	0.1682	−0.0013	0.049*	
C4	0.3207 (4)	0.1809 (2)	0.10433 (19)	0.0302 (8)	
C5	0.5128 (4)	0.1365 (3)	0.3852 (2)	0.0322 (8)	
C6	0.3261 (5)	0.0392 (3)	0.4279 (3)	0.0478 (11)	
H6	0.2938	−0.0021	0.4657	0.057*	
C7	0.2193 (5)	0.0569 (3)	0.3644 (2)	0.0448 (10)	
H7	0.1152	0.0271	0.3563	0.054*	
C8	0.2688 (5)	0.1201 (3)	0.3123 (2)	0.0411 (9)	
H8	0.1956	0.1349	0.2680	0.049*	

### Atomic displacement parameters (Å^2^)

**Table:**

	*U*^11^	*U*^22^	*U*^33^	*U*^12^	*U*^13^	*U*^23^
Mn1	0.0302 (4)	0.0301 (4)	0.0306 (4)	0.000	0.0034 (3)	0.000
I1	0.03561 (15)	0.03743 (15)	0.04207 (16)	0.00468 (11)	0.01272 (11)	0.00650 (11)
N1	0.0328 (16)	0.0308 (16)	0.0310 (16)	0.0042 (13)	0.0041 (13)	−0.0004 (13)
N2	0.0335 (17)	0.0430 (18)	0.0345 (18)	0.0038 (14)	0.0031 (14)	−0.0054 (14)
N3	0.0395 (19)	0.049 (2)	0.044 (2)	−0.0127 (16)	0.0022 (16)	0.0159 (16)
N4	0.0351 (17)	0.0333 (16)	0.0356 (18)	−0.0060 (14)	0.0023 (14)	−0.0003 (14)
C1	0.035 (2)	0.036 (2)	0.038 (2)	0.0031 (17)	0.0107 (16)	−0.0092 (17)
C2	0.030 (2)	0.051 (2)	0.045 (2)	0.0079 (18)	0.0051 (17)	0.003 (2)
C3	0.035 (2)	0.051 (2)	0.035 (2)	−0.0030 (18)	0.0010 (17)	−0.0035 (18)
C4	0.036 (2)	0.0299 (18)	0.0252 (18)	0.0017 (15)	0.0047 (15)	0.0020 (15)
C5	0.0315 (19)	0.0300 (18)	0.036 (2)	−0.0062 (15)	0.0064 (16)	−0.0026 (16)
C6	0.048 (3)	0.045 (2)	0.051 (3)	−0.013 (2)	0.009 (2)	0.013 (2)
C7	0.035 (2)	0.042 (2)	0.056 (3)	−0.0135 (18)	0.004 (2)	0.001 (2)
C8	0.038 (2)	0.042 (2)	0.042 (2)	−0.0096 (18)	−0.0002 (18)	0.0008 (18)

### Geometric parameters (Å, °)

**Table:**

Mn1—N4^i^	2.289 (3)	N4—C5	1.338 (5)
Mn1—N4	2.289 (3)	C1—C2	1.383 (5)
Mn1—N1	2.305 (3)	C1—H1	0.9500
Mn1—N1^i^	2.305 (3)	C2—C3	1.381 (6)
Mn1—I1	2.8410 (5)	C2—H2	0.9500
Mn1—I1^i^	2.8410 (5)	C3—H3	0.9500
N1—C1	1.334 (5)	C4—C5^i^	1.498 (5)
N1—C4	1.339 (4)	C5—C4^i^	1.498 (5)
N2—C3	1.325 (5)	C6—C7	1.361 (6)
N2—C4	1.332 (5)	C6—H6	0.9500
N3—C5	1.315 (5)	C7—C8	1.378 (6)
N3—C6	1.347 (5)	C7—H7	0.9500
N4—C8	1.332 (5)	C8—H8	0.9500
			
N4^i^—Mn1—N4	84.55 (16)	N1—C1—H1	119.5
N4^i^—Mn1—N1	71.78 (10)	C2—C1—H1	119.5
N4—Mn1—N1	90.72 (11)	C3—C2—C1	117.1 (4)
N4^i^—Mn1—N1^i^	90.72 (11)	C3—C2—H2	121.4
N4—Mn1—N1^i^	71.78 (10)	C1—C2—H2	121.4
N1—Mn1—N1^i^	156.60 (15)	N2—C3—C2	122.9 (4)
N4^i^—Mn1—I1	86.90 (8)	N2—C3—H3	118.5
N4—Mn1—I1	164.40 (8)	C2—C3—H3	118.5
N1—Mn1—I1	98.98 (7)	N2—C4—N1	126.1 (3)
N1^i^—Mn1—I1	95.35 (7)	N2—C4—C5^i^	117.2 (3)
N4^i^—Mn1—I1^i^	164.40 (8)	N1—C4—C5^i^	116.7 (3)
N4—Mn1—I1^i^	86.90 (8)	N3—C5—N4	126.6 (3)
N1—Mn1—I1^i^	95.35 (7)	N3—C5—C4^i^	117.4 (3)
N1^i^—Mn1—I1^i^	98.98 (7)	N4—C5—C4^i^	116.0 (3)
I1—Mn1—I1^i^	104.19 (3)	N3—C6—C7	122.0 (4)
C1—N1—C4	117.1 (3)	N3—C6—H6	119.0
C1—N1—Mn1	125.8 (2)	C7—C6—H6	119.0
C4—N1—Mn1	117.0 (2)	C6—C7—C8	117.2 (4)
C3—N2—C4	115.8 (3)	C6—C7—H7	121.4
C5—N3—C6	116.2 (3)	C8—C7—H7	121.4
C8—N4—C5	115.7 (3)	N4—C8—C7	122.2 (4)
C8—N4—Mn1	125.9 (3)	N4—C8—H8	118.9
C5—N4—Mn1	117.7 (2)	C7—C8—H8	118.9
N1—C1—C2	120.9 (3)		
			
N4^i^—Mn1—N1—C1	176.2 (3)	Mn1—N1—C1—C2	−177.5 (3)
N4—Mn1—N1—C1	92.1 (3)	N1—C1—C2—C3	0.3 (6)
N1^i^—Mn1—N1—C1	132.8 (3)	C4—N2—C3—C2	0.5 (6)
I1—Mn1—N1—C1	−100.1 (3)	C1—C2—C3—N2	−0.7 (6)
I1^i^—Mn1—N1—C1	5.2 (3)	C3—N2—C4—N1	0.1 (6)
N4^i^—Mn1—N1—C4	−1.6 (2)	C3—N2—C4—C5^i^	−179.7 (3)
N4—Mn1—N1—C4	−85.6 (3)	C1—N1—C4—N2	−0.5 (5)
N1^i^—Mn1—N1—C4	−44.9 (2)	Mn1—N1—C4—N2	177.4 (3)
I1—Mn1—N1—C4	82.1 (2)	C1—N1—C4—C5^i^	179.4 (3)
I1^i^—Mn1—N1—C4	−172.6 (2)	Mn1—N1—C4—C5^i^	−2.7 (4)
N4^i^—Mn1—N4—C8	−90.9 (3)	C6—N3—C5—N4	0.8 (6)
N1—Mn1—N4—C8	−19.2 (3)	C6—N3—C5—C4^i^	−179.7 (4)
N1^i^—Mn1—N4—C8	176.6 (3)	C8—N4—C5—N3	−1.6 (6)
I1—Mn1—N4—C8	−148.0 (3)	Mn1—N4—C5—N3	169.7 (3)
I1^i^—Mn1—N4—C8	76.1 (3)	C8—N4—C5—C4^i^	178.9 (3)
N4^i^—Mn1—N4—C5	98.8 (3)	Mn1—N4—C5—C4^i^	−9.8 (4)
N1—Mn1—N4—C5	170.5 (3)	C5—N3—C6—C7	1.2 (7)
N1^i^—Mn1—N4—C5	6.3 (3)	N3—C6—C7—C8	−2.3 (7)
I1—Mn1—N4—C5	41.8 (5)	C5—N4—C8—C7	0.3 (6)
I1^i^—Mn1—N4—C5	−94.2 (3)	Mn1—N4—C8—C7	−170.1 (3)
C4—N1—C1—C2	0.2 (5)	C6—C7—C8—N4	1.5 (7)

Symmetry codes: (i) −*x*+1, *y*, −*z*+1/2.

### Hydrogen-bond geometry (Å, °)

**Table:**

*D*—H···*A*	*D*—H	H···*A*	*D*···*A*	*D*—H···*A*
C6—H6···N3^ii^	0.95	2.60	3.151 (5)	117.
C6—H6···N2^iii^	0.95	2.62	3.554 (5)	166.
C7—H7···I1^iv^	0.95	2.97	3.917 (4)	173.

Symmetry codes: (ii) −*x*+1, −*y*, −*z*+1; (iii) *x*, −*y*, *z*+1/2; (iv) −*x*+1/2, *y*−1/2, −*z*+1/2.
